# Colloid Chemistry

**DOI:** 10.3390/gels4030064

**Published:** 2018-07-23

**Authors:** Clemens K. Weiss

**Affiliations:** Department of Life Sciences and Engineering, University of Applied Sciences Bingen, Berlinstrasse 109, 55411 Bingen, Germany; c.weiss@th-bingen.de; Tel.: +49-6721-409270

Colloid Chemistry has always been an integral part of several chemical disciplines. Ranging from preparative inorganic chemistry to physical chemistry, researchers have always been fascinated in the dimensions and the possibilities colloids offer. Since the advent of nanotechnology and analytical tools, which have evolved across recent decades, colloid chemistry or “nano-chemistry” has become essential for high-level research in various disciplines. 

The contributions to this Special Issue cover most of the important aspects: choice, design and synthesis of building blocks; preparation and modification of gel and colloidal structures; analysis and application as well as the study of physical and physicochemical phenomena. Most importantly, the contributions connect these aspects, relate them and present a comprehensive overview.

Small molecules can act as gelators as well as polymers or colloids. The chemical structure of these building blocks defines the interactions between them and thus the structure and properties of the macroscopic material. Malo de Molina et al. [1] present a comprehensive review of colloidal structures generated by self-assembly of amphiphilic molecules. Assemblies of small molecule surfactants as well as amphiphilic polymers in water can form hydrogels. The resulting morphologies are discussed and routes to gelation are described. Latxague et al. [2] show a synthetic approach towards a bolaamphiphile based on structures found in living nature. Based on thymidine and a saccharide moiety two hydrophilic groups are linked symmetrically to a hydrophobic spacer via click chemistry. Carbamate groups contribute to gel properties with supramolecular hydrogen bonding.

Gels obtained from polysaccharide or other natural polymers were reviewed by Karoyo and Wilson [3] and del Valle et al. [4]. These materials hold great promise for application in food, cosmetic, biomedicine, pharmaceutical sciences but also for technical applications as e.g., catalysis. Tailored properties are required for all of the mentioned applications, thus the possibility to control properties such as stability, dimension and response to external stimuli is paramount. Karoyo and Wilson discuss supramolecular interactions leading to host-guest systems and present methods for structural characterization. In addition to the biomedical prospects of peptide-based hydrogels, del Valle et al. point our approaches to molecular imprinting and 3D bioprinting.

The formation of gels from colloidal structures is presented by van Doorn et al. [5] and by Hijnen and Clegg [6]. While van Doorn et al. studied the behavior of surface functionalized spherical nanoparticles, Hijnen and Clegg studied the behavior of sphero-cylinders in dispersion. Van Doorn et al. functionalized the surface of colloidal particles with a surface-initiated Atomic Transfer Radical Polymerisation (ATRP) technique. They used *N*-isopropylacrylamide (NIPAAM) for generating a thermoresponsive polymer corona on the particles. The gelation and gel properties were studied in dependence of grafting density, chain length and temperature. It is shown how sophisticated particle design allows for the controlling of macroscopic bulk properties. Hijnen and Clegg point out the interesting features that non-spherical particles exhibit in dispersions of various volume fractions. They present trigger-induced phase separation as a convenient tool for the generation of percolating particle networks. 

Two dimensional structures created from colloidal particles are presented by Bähler et al. [7]. Colloidal monolayers with tunable interparticle spacing present valuable starting materials for several applications, such as the generation of plasmonic substrates. There is, however, the difficulty of removing such monolayers from the interface without disturbing their position and order. The contribution presents three ways of embedding the monolayer in a polymeric film, creating a colloid containing membrane, which can easily be removed from the interface.

Non-spherical particles are also used by Cohen et al. [8]. The authors prepared suspensions of fluorescently labelled photo-crosslinkable polymethylmethacrylate (PMMA) spheres. The dynamics and structure of these suspensions were thoroughly studied by dynamic light scattering (DLS) and the recently developed technique of confocal differential dynamic microscopy. The same techniques were used for the study of ellipsoidal particles, which were created by stretching the above mentioned PMMA spheres. 

The preparation and application of spherical assemblies, so-called supraparticles, aided by superhydrophobic surfaces, were reviewed by Sperling and Gradzielski [9]. They point out that such complex structures can conveniently be prepared, when dispersions are evaporated in a controlled manner, ideally on superhydrophobic surfaces. The authors comprehensively present and evaluate the enormous possibilities of the technique for controlling shape, interior and functionalities. Finally, they outline several potential applications ranging from biomedical applications to self-propelled particles. 

Understanding how the structure of colloids or gels affects the microscopic or macroscopic properties is essential for rational material design. Starndman and Zhu [10] show how the performance and the properties of self-healing dynamic gel structures is affected by supramolecular interactions in gel materials and in which way the tailoring of interaction controls the properties. The authors also point towards potential applications of these materials e.g., in biomedicine. Transport phenomena in gel networks are reviewed by Tokita [11]. Regarded as solvent stabilized by a polymeric network, small molecule transport is governed by diffusion, viscosity, and the solvent flow as well as by the resistance imposed by the polymer network.

Strzelczyk et al. [12] used modified poly(ethylene glycol) (PEG)-based microgels for studying adhesive processes and quantifying adhesion energies. The functionalized microgels were brought into contact with functionalized glass slides. The complementary functionalization lead to stronger adhesion as without functionalization. The magnitude of adhesion was calculated with the contact areas, obtained by interferometric measurements. Two examples from biomedicine, antibody recognition, and laundry, release of soil polymers, showed that this platform is a versatile and convenient sensor for measuring adhesion properties.

The breadth of the contributions underlines the significance of colloid chemistry for a variety of disciplines. Enjoy reading!
